# Intracranial pulse pressure waveform analysis using the higher harmonics centroid

**DOI:** 10.1007/s00701-021-04958-1

**Published:** 2021-08-13

**Authors:** Agnieszka P. Zakrzewska, Michał M. Placek, Marek Czosnyka, Magdalena Kasprowicz, Erhard W. Lang

**Affiliations:** 1 Brain Physics Laboratory, Division of Neurosurgery, Department of Clinical Neurosciences, University of Cambridge, Cambridge, UK; 2 Department of Biomedical Engineering, Faculty of Fundamental Problems of Technology, Wroclaw University of Science and Technology, Wybrzeze Wyspiańskiego 27, 50-370 Wroclaw, Poland; 3 Neurosurgical Associates, Red Cross Hospital, Kassel, Germany; 4 Faculty of Medicine, Department of Neurosurgery, Georg-August-Universität, Göttingen, Germany; 5 Institute of Electronic Systems, Warsaw University of Technology, Warsaw, Poland

**Keywords:** Traumatic brain injury, Intracranial pressure, Intracranial pressure pulse waveform, Plateau waves, ICP monitoring

## Abstract

**Background:**

The pulse waveform of intracranial pressure (ICP) is its distinctive feature almost always present in the clinical recordings. In most cases, it changes proportionally to rising ICP, and observation of these changes may be clinically useful. We introduce the higher harmonics centroid (HHC) which can be defined as the center of mass of harmonics of the ICP pulse waveform from the 2nd to 10th, where mass corresponds to amplitudes of these harmonics. We investigate the changes in HHC during ICP monitoring, including isolated episodes of ICP plateau waves.

**Material and methods:**

Recordings from 325 patients treated between 2002 and 2010 were reviewed. Twenty-six patients with ICP plateau waves were identified. In the first step, the correlation between HHC and ICP was examined for the entire monitoring period. In the second step, the above relation was calculated separately for periods of elevated ICP during plateau wave and the baseline.

**Results:**

For the values averaged over the whole monitoring period, ICP (22.3 ± 6.9 mm Hg) correlates significantly (*R* = 0.45, *p* = 0.022) with HHC (3.64 ± 0.46). During the ICP plateau waves (ICP increased from 20.9 ± 6.0 to 53.7 ± 9.7 mm Hg, *p* < 10^−16^), we found a significant decrease in HHC (from 3.65 ± 0.48 to 3.21 ± 0.33, *p* = 10^−5^).

**Conclusions:**

The good correlation between HHC and ICP supports the clinical application of pressure waveform analysis in addition to the recording of ICP number only. Mean ICP may be distorted by a zero drift, but HHC remains immune to this error. Further research is required to test whether a decline in HHC with elevated ICP can be an early warning sign of intracranial hypertension, whether individual breakpoints of correlation between ICP and its centroid are of clinical importance.

## Introduction

Intracranial compliance (ICC) describes the capacity of intracranial compartments to contain an additional volume with no significant change in intracranial pressure (ICP) [30]. ICC is sometimes considered to be an earlier and more sensitive indicator of secondary brain insults and neurological deterioration than “classic” ICP mean value itself. Early works reported that changes in ICP pulse waveform indicate changes in ICC, which may be clinically useful above the traditionally considered ICP value itself [3, 25, 28, 29, 33, 34].

**Fig. 1:**
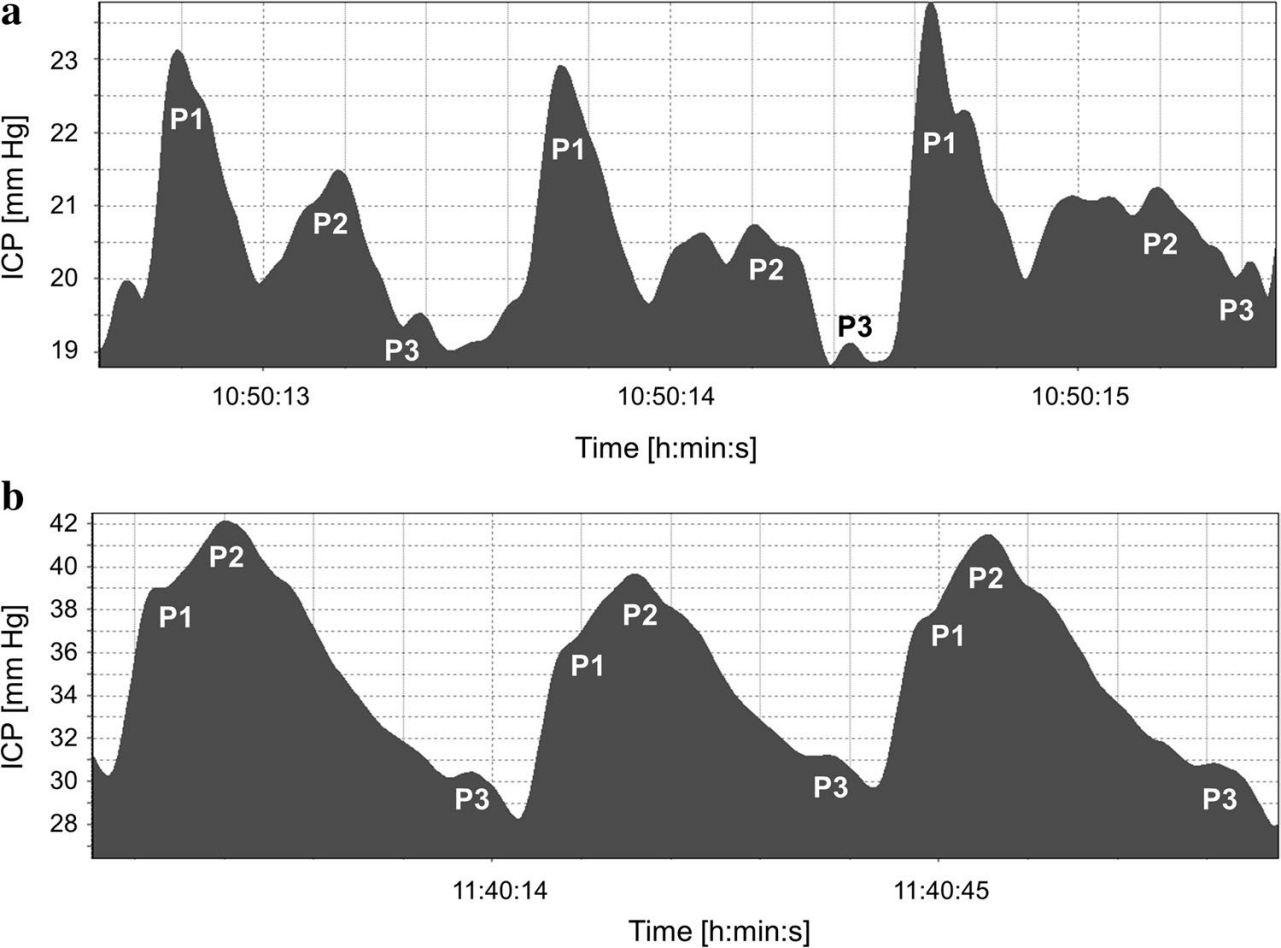
Illustrative patterns of intracranial pressure (ICP) pulse waveform. **a** Three components of ICP pulse waves at normal ICP and good compliance. In some cases, P2 has a biphasic appearance. **b** Three components of the ICP pulse waveform at high ICP and decreased compliance

The pulse waveform of ICP is reflected by the fluctuations of the cerebrospinal fluid generated by the systole and diastole of the cardiac cycle. Visual inspection of the ICP pulse waveform usually reveals three distinct peaks: P1, percussion wave; P2, tidal wave; and P3, dicrotic wave [8, 18] (Fig. 1). They are also termed “component waves” [20]. Despite intensive research on the morphology of the ICP pulse waveform over years, the origin of the peaks remains unclear. Recent investigation with the phase-contrast magnetic resonance imaging found that both rapid intracranial volume changes and transmission of the arterial blood pressure (ABP) shape the ICP curve [2, 4, 36]. Morphology of ICP waveform is important because P1 and P2 relation describes brain compliance. Under normal physiological conditions, P1 exceeds P2 and P2 exceeds P3 (Fig. 1a), indicating good ICC. Early reports already show and describe that the shape of the ICP waveform changes with rising ICP [1, 23], indicating depletion in ICC. It was reported that “the spontaneous increase in intracranial pressure (ICP) is accompanied by a disproportionate elevation of the cerebrospinal fluid pulse wave components P2 and P3, resulting in changes of the shape of the pulse wave. It first becomes rounded and, at higher ICP values, it acquires a pyramidal shape” [8] (Fig. 1b).

**Fig. 2:**
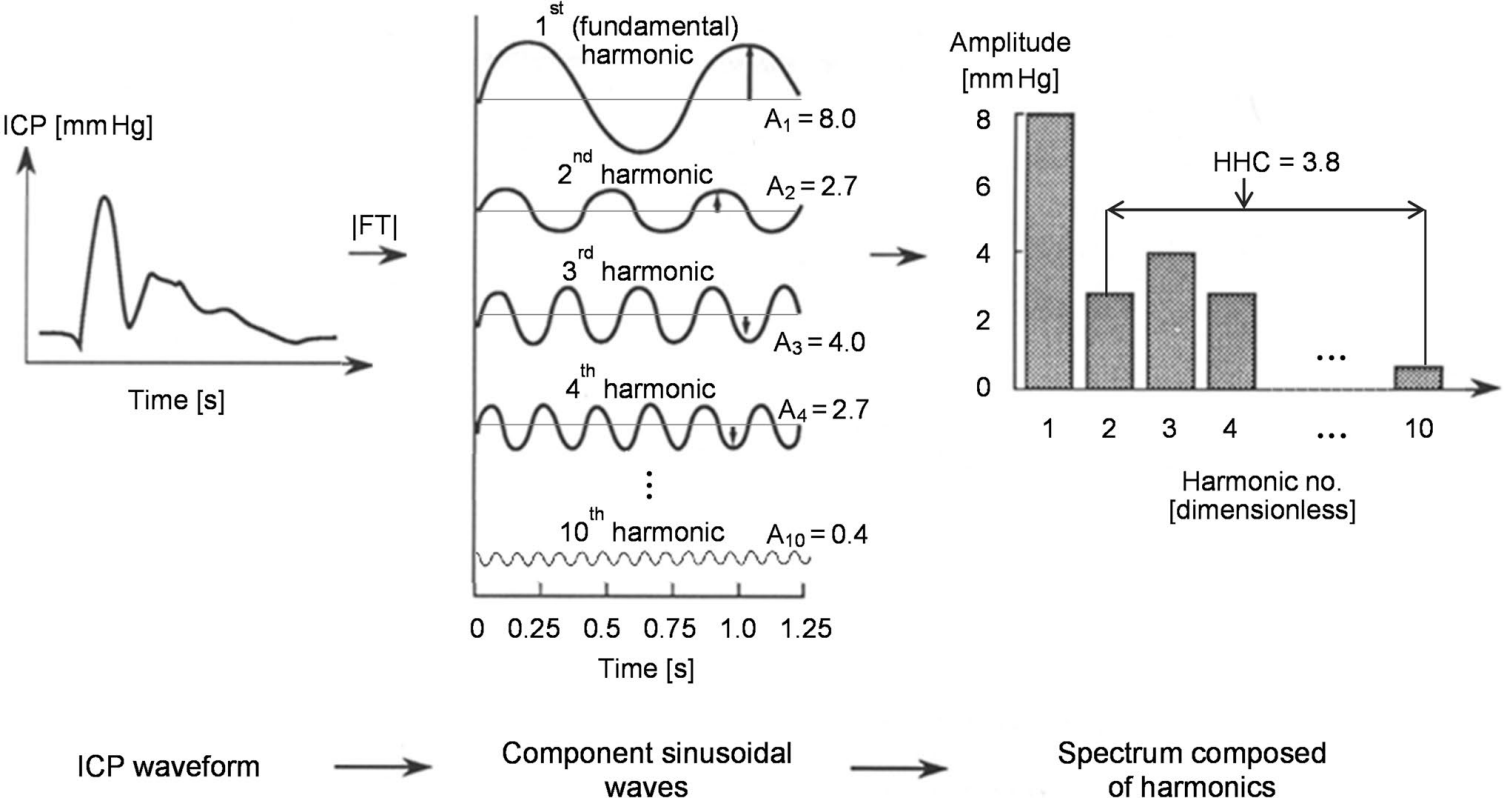
The way of calculating the higher harmonics centroid (HHC). Fourier transform (FT) decomposes a function of time, e.g., the intracranial pressure (ICP) waveform, into separate sinusoidal waves, each with a different frequency and amplitude. Here, fundamental frequency corresponds to the pulse. In the presented analysis, the HHC is the amplitude-weighted average of ICP pulse waveform harmonics from 2nd to 10th expressed as consecutive integers from 2 to 10. The HHC is dimensionless

Other than classifying ICP pulse waveform based on visual inspection [21], it is possible to calculate various metrics of higher harmonics distortion of the pulse wave. Previously, a higher frequency centroid (HFC) has been introduced [7, 13, 22, 31]. It was demonstrated that a rise in centroid correlated with changes in ICP [31] and was supposed to signify transient intracranial hypertension [13]. It was also shown that a decrease in HFC was associated with refractory intracranial hypertension [13]. However, this early observation has never been confirmed in the practice of neurosurgery and neurocritical care worldwide. We introduced another metric of the ICP pulse waveform which is the higher harmonics centroid (HHC) [5, 35]. HHC is the center of mass of ICP pulse waveform harmonics from the 2nd to 10th expressed as consecutive integers from 2 to 10, where mass corresponds to amplitudes of these harmonics (Fig. 2). Other harmonics have also been assessed in previous studies [11, 12, 27, 38].

These concepts are potentially attractive because “continuous monitoring of intracranial compliance by computerized analysis of the ICP waveform may provide an earlier warning of neurological decompensation than ICP per se and, unlike pressure–volume index (PVI), does not require volumetric manipulation of intracranial volume” [31].

In this study, we aim to investigate the correlations between HHC and ICP, ABP, cerebral perfusion pressure (CPP), heart rate (HR), and PRx, the cerebral pressure reactivity index [14] for (1) the entire monitoring time performed in patients after traumatic brain injury and (2) during isolated ICP plateau waves and at their corresponding baseline episodes.

## Material and methods

The study comprised patients after traumatic brain injury (TBI), treated in the Neurosciences Critical Care Unit (NCCU) in Addenbrooke’s Hospital, Cambridge, UK, from 2002 to 2010. The use of the data for anonymous audit and publication was approved by the institutional Research Ethics Committee (29 REC 97/291). Informed consent for this retrospective analysis was waived.

We selected 26 patients with 86 ICP plateau waves from the 325-patient database of multimodality brain monitoring. Table 1 provides a summary of patient demographics. All patients were sedated and mechanically ventilated receiving standard neurocritical care at Cambridge University Hospitals, with details provided elsewhere [15, 37].

**Table 1:** Patient demographics and clinical information

Factor	No of patients
Initial CGS	
3	6
4	1
5	2
6	4
7	1
8	2
10	1
11	1
12	1
13	1
14	1
n/a	5
Age	38 ± 13 years (range 17–72 years)
Female	8
Male	18
Outcome at 6 months	
Good recovery	9
Moderate disability	3
Severe disability	2
Persistent vegetative state	1
Dead	11

ICP was monitored using an intraparenchymal micro-sensor (Codman ICP MicroSensor, Codman & Shurtleff, Raynham, MA) inserted in the frontal white matter via a cranial access device (Technicam, Newton Abbot, UK). Zeroing was standard and performed at the time of implantation of the ICP sensor—wet transducer tip zeroed against the atmospheric pressure. ABP was measured through a line inserted into the radial or femoral artery with a zero calibration at the level of the right atrium. All patients were positioned with head elevation to 30° during the monitoring period, as per standard intensive care protocols for conservative management of severe head injury [24]. All individuals had high-resolution ICP and ABP signals recorded on a bedside computer, with a frequency of 50 Hz. The ICM + software (Cambridge Enterprise, Cambridge, UK, https://icmplus.neurosurg.cam.ac.uk) was used for online data recording. Computerized data monitoring which supports critical care management is a standard clinical practice in Cambridge.

HHC was calculated in the following way. First, ICP waveform was divided into 10-s-long non-overlapping segments. Fourier transform was applied to decompose the segment of ICP into separate sinusoids, each with a different frequency and amplitude. The spectrum generated by the Fourier transform shows the amplitudes of these sinusoids plotted against their respective frequencies. The first harmonic (fundamental frequency) corresponding to the pulse (heart rate) was found as a frequency within the range of 40–180 beats per minute for which the amplitude was maximal. From the amplitude spectrum, we extracted only the fundamental component and its higher harmonics. Our amplitude spectrum was plotted against harmonic numbers instead of frequencies (Fig. 2). This approach was chosen to reduce the influence of changes in heart rate on the ICP amplitude spectrum. The higher harmonics centroid (HHC) was defined as the amplitude-weighted average of ICP pulse waveform harmonics from the 2nd to 10th expressed as consecutive integers from 2 to 10.

**Fig. 3:**
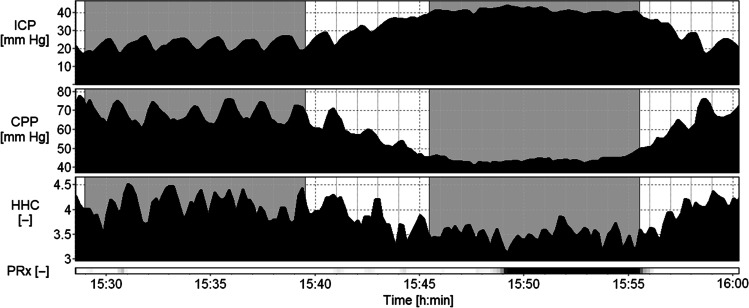
Illustrative smoothed time course of selected parameters during baseline episode (first shaded area) and plateau wave (second shaded area). ICP, intracranial pressure; CPP, cerebral perfusion pressure; HHC, higher harmonics centroid; PRx, cerebrovascular reactivity index. PRx is also shown as a risk chart where black corresponds to PRx ≥ 0.4. Symbol [–] denotes dimensionless unit

Plateau waves were identified during post-recording offline analysis and inspection of the data. The identification criteria were an ICP elevation above 35 mm Hg, an average relative ICP increase of greater than or equal to 15 mm Hg, matched by a CPP decrease of greater than or equal to 10 mm Hg [16, 32]. These criteria were based on the original definition of a plateau wave given in the experimental work [32] and further adapted to clinical conditions where increases in ICP are usually actively treated. For each plateau wave, an individual baseline episode preceding the plateau wave was extracted. A stable ICP level of a duration of 15–20 min was selected. ICP increase of at least 15 mm Hg and CPP decrease of at least 10 mm Hg during plateau wave was checked against the average ICP and CPP obtained from the baseline period (Fig. 3).

### Statistical analysis

The relationships between HHC and the other parameters were investigated using multivariate regression analysis. The patient’s identification number was included in the model using binary dummy variables recoding. Partial correlation coefficients, which take into account repeated observations, were calculated [6]. Moreover, values of the parameters calculated from multiple plateau waves and their corresponding baseline episodes registered in one patient were averaged separately, yielding baseline and plateau waves values of these parameters. Subsequently, these mean baseline values with mean plateau values were compared using paired t-test (assumption of normality was not rejected by the Shapiro–Wilk test, *p* > 0.1 for all variables). To identify a breaking point on ICP-HHC characteristics, a scatter plot and an error bar chart (mean ± confidence interval) were obtained for each patient’s individual recording as well as for pooled data.

## Results

Table 2 provides physiological values and calculated variables averaged over the entire monitoring time, as well as differences in analyzed parameters during baseline episodes and plateau waves. HHC decreases significantly during plateau waves. For values averaged over the whole monitoring time, HHC correlates positively with ICP, ABP, and HR but it does not correlate with either CPP or PRx. See Table 3 for detailed results of the correlation analysis. During plateau waves, HHC correlates negatively with ICP, ABP, and PRx. Interestingly, it does not correlate with CPP. No significant correlation with heart rate is seen during the plateau wave.

**Table 2:** Physiological values and calculated variables during the entire monitoring time, baseline episodes, and peak plateau waves

	ICP [mm Hg]	CCP [mm Hg]	ABP [mm Hg]	PRx [–]	HR [beats/min]	HHC [–]
Whole	22.3 ± 6.9	74.9 ± 7.0	96.5 ± 9.4	0.03 ± 0.19	75.5 ± 15.0	3.64 ± 0.46
Baseline	20.9 ± 6.0	77.6 ± 10.6	98.5 ± 12.8	0.02 ± 0.18	75.2 ± 16.3	3.65 ± 0.48
Plateau	53.7 ± 9.7	48.8 ± 9.9	102.5 ± 13.8	0.51 ± 0.21	78.4 ± 17.4	3.21 ± 0.33
*p*-value Baseline vs. plateau	10^−16^	10^−16^	0.021	10^−10^	0.14	10^−5^

Values are expressed as mean ± SD and compared with paired t-test (baseline vs. plateau). *ICP* intracranial pressure, *CPP* cerebral perfusion pressure, *ABP* arterial blood pressure, *PRx* cerebrovascular reactivity index, *HR* hear rate, *HHC* higher harmonics centroid. Symbol [–] denotes a dimensionless unit

**Table 3:** Correlation analysis of HHC and other variables on three stages of recording: whole recording, baseline before plateau wave and during plateau wave

		Whole	Baseline	Plateau
ICP [mm Hg]	*R*_p_	**0.45**	**–0.31**	**–0.65**
	*p*	**0.022**	**0.013**	** < 10**^**−8**^
CPP [mm Hg]	*R*_p_	0.34	0.18	0.19
	*p*	0.094	0.16	0.13
ABP [mm Hg]	*R*_p_	**0.5**	0.06	**–0.43**
	*p*	**0.009**	0.65	**0.0005**
PRx [–]	*R*_p_	–0.23	0.12	–0.41
	*p*	0.26	0.36	**0.001**
HR [beats/min]	*R*_p_	**–0.73**	**–0.35**	0.09
	*p*	**0.00004**	**0.005**	0.51

*ICP* intracranial pressure, *CPP* cerebral perfusion pressure, *ABP* arterial blood pressure, *PRx* cerebrovascular reactivity index, *HR* heart rate, *HHC* higher harmonics centroid, *R*_p_ partial correlation coefficient, *p p*-value. Statistically significant correlations are shown in bold

**Fig. 4:**
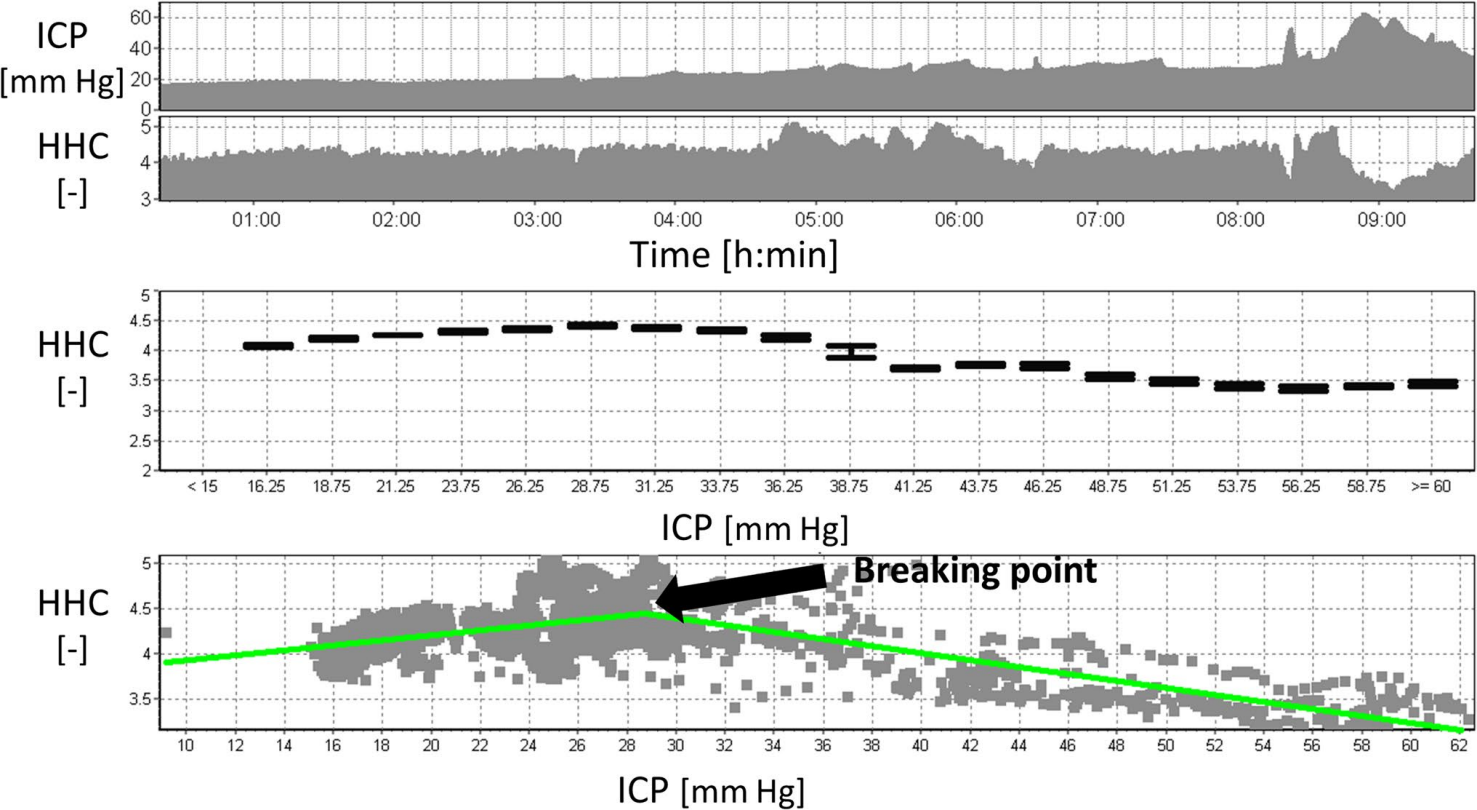
Illustrative smoothed time course of ICP and HHC, the HHC error bar chart and the ICP-HHC scatter diagram plotted from a 10-h-long recording performed in a single patient. A decrease in HHC (a breakpoint) is observed at the ICP of approximately 31 mmHg. ICP, intracranial pressure; HHC, higher harmonics centroid. Symbol [–] denotes a dimensionless unit

**Fig. 5:**
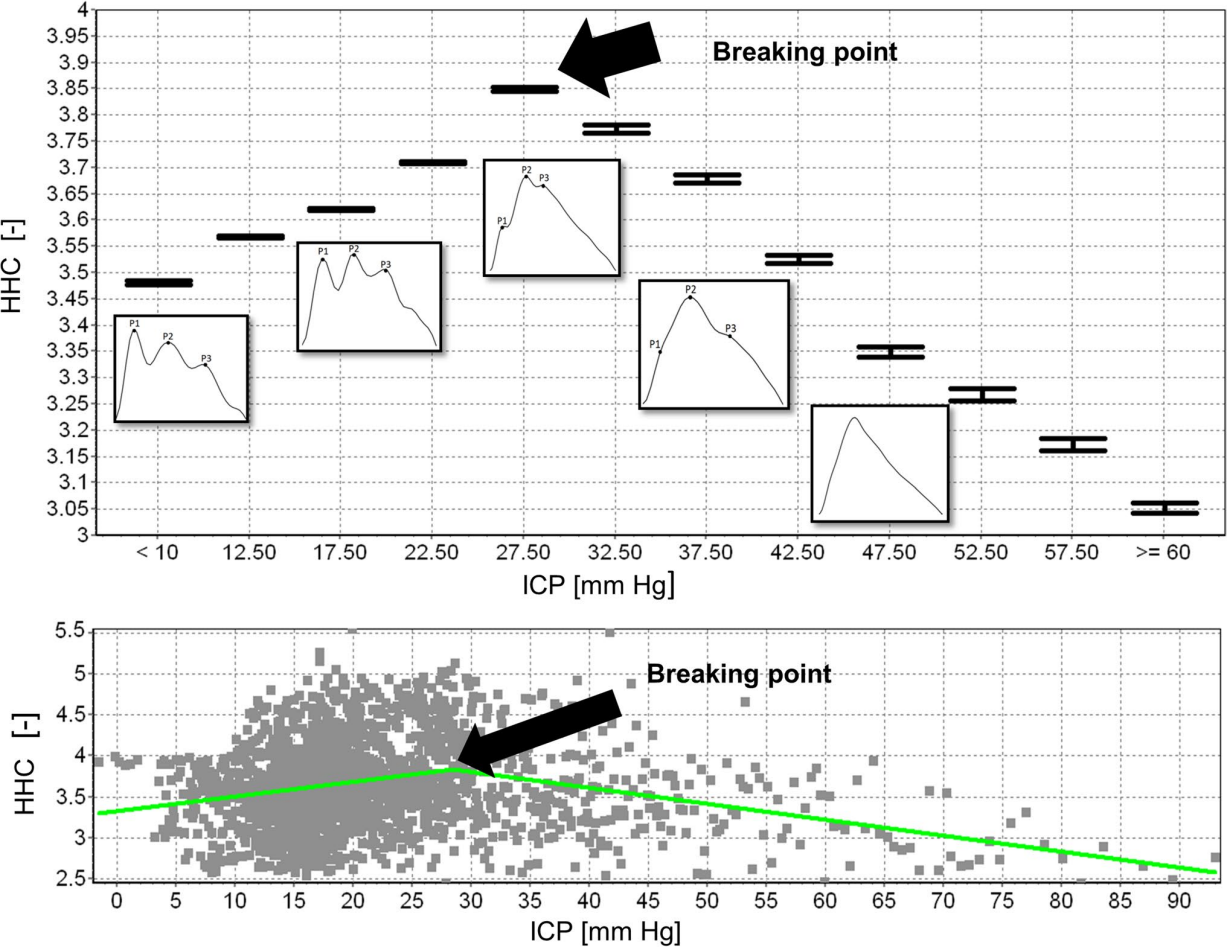
The HHC error bar chart (upper panel) and the ICP-HHC scatter diagram (bottom panel) plotted for pooled data (26 ICP recordings). HHC increases with rising ICP up to approximately 27.5 mm Hg (a breaking point) and then decreases with a further ICP elevation. The change in the shape of the ICP pulse waveform (from the typical three-peak appearance to triangular shape) initially causes the HHC to increase and then, after crossing the breakpoint, the HHC eventually decreases due to the lower content of higher frequencies. ICP, intracranial pressure; HHC, higher harmonics centroid. Symbol [–] denotes a dimensionless unit

An exemplary HHC error chart and a scatter plot obtained from a 10-h recording of ICP performed in a single patient are presented in Fig. 4. The average value of the ICP at which the breaking point occurs in individual ICP-HHC characteristics is 30.7 ± 6.1 mm Hg. The error bar chart and the ICP-HHC scatter diagram plotted for pooled data (Fig. 5) show a gradual increase in HHC with rising ICP up to approximately 27.5 mm Hg and then a decline with a further elevation of the pressure.

## Discussion

This study shows that patient-averaged HHC correlates well with ICP outside of plateau waves and confirms findings from previous reports [26, 31]. This supports the clinical utility of using computerized ICP pulse waveform analysis, in addition to the mere ICP value, to indicate states of compromised cerebral function after TBI, which has been demonstrated to have a negative prognostic impact [31]. On the other hand, our data analysis reveals that HHC decreases during plateau waves. Only one paper so far has reported on centroid analysis in a subgroup of TBI patients who developed refractory intracranial hypertension [13]. Previously reported data does not include centroid analysis during plateau waves. Berdyga et al. report a decrease in HHC during the controlled rise in ICP in infusion test performed in patients suffering from hydrocephalus [5].

The main goal of this study was to investigate if the HHC indicates clinically relevant intracranial hypertension. This was answered affirmatively. An outcome correlation was not included, because the study comprised patients with plateau waves individually selected from a large database, resulting in a small subgroup of patients.

### Comparison with previous studies

This is the first report assessing the correlation of HHC with monitored parameters of brain physiology. Thus, there are no comparative studies examining these pulse wave metrics to date. However, research in the computerized analysis of ICP pulse waveform by a group from the Department of Neurosurgery at Baylor College of Medicine, Houston, USA, focused on another centroid, namely HFC. It is the power-weighted average frequency within the 4- to 15-Hz band of the ICP power density spectrum [13, 22, 31]. Although it is a different metric, it presents a similar drift with rising ICP. Other frequency bands have been investigated before by Bray et al.[7].

Robertson et al. [31] did not report their ICP values, which does not enable a close-up comparison with our findings. We speculate, however, that their ICP values were lower than ours because they reported that ICP > 25 mm Hg was considered a neurosurgical emergency and aggressive treatment was initiated. Our mean ICP for the entire monitoring time was 22 ± 7 mm Hg with a range from 11 to 39 mm Hg. Robertson et al. might have encountered a decrease in HFC with rising ICP if their ICP values had been higher.

The second paper from the Houston Group 6 years later, using a different dataset, includes a subgroup of patients who go on to develop refractory intracranial hypertension [13]. One of their examples shows a gradual HFC decrease from slightly above 8 to about 7.4 Hz when they compared three patient groups with ICP < 20, ICP 20–30, and ICP > 30 mmHg. These findings are also consistent with our report.

### Clinical relevance

It is a daily clinical observation that ICP pulse waveforms at a given ICP value differ within and between patients. This may have many causes including patient age, distribution of intra- or extracellular brain oedema/swelling, cerebral blood flow and cerebrovascular autoregulatory function, degree of hyperventilation, time after injury, and inflammatory reactions.

It is also known that the typical three-peak appearance of the ICP pulse wave gets lost with increasing pulse amplitude during rising ICP [8, 25], turning the wave eventually into a triangular shape. This change in the shape causes a decrease in the higher frequencies components, resulting in a decline in the HHC (Fig. 5).

We are not aware of any study that identified breakpoints in the ICP-HHC correlation. We showed that such a breakpoint exists and its occurrence may be a warning sign with a need to decrease ICP or a sign of improvement in case of an increasing HHC with decreasing ICP. This topic may spark some research interest based on this study and even expand the armamentarium of modalities in clinical brain monitoring in the future.

### Higher harmonics centroid

The possible advantage of the HHC over the HFC is its independence of heart rate. Potential increases in the HFC can be induced by increases in the HR, which can occur with rising ICP and during plateau waves [13]. This effect should be theoretically less apparent in the HHC analysis. Unlike the HFC, HHC is dimensionless, takes into account harmonics’ number instead of powers, and ignores components of the ICP wave that may appear between higher harmonics.

Our study shows that the HHC was found to be lower during plateau waves when compared to the entire monitoring time. This drop additionally occurs between baseline and peak plateau waves. HR increases in the presented material were not large enough to match concerns of compounding with HHC.

To the knowledge of the authors, there are no publications on the analysis of ICP pulse waveform with the use of the HHC. One study in the neurosurgical literature reported that only harmonics nos. 1 to 9 of the pulse pressure wave were of significant size. This publication further concluded that “the close correlation between the amplitude of the 1^st^ harmony and the pulse amplitude is mainly composed of low-frequency components, because the amplitude of the 1st harmony is the most significant in the Fourier spectrum of the PPW (pressure pulse wave)” [12]. This is, at least to a certain degree, reflected in our analysis, with HHC values of 3.64 ± 0.45 for the entire monitoring time.

The second article which we found had examined some harmonics of the ICP waveform in relation to different ICP values [13]. It reports that the amplitudes of the fundamental wave (FW), harmonics 2nd, 4th, and 5th, were elevated during the peak of ICP. The increases in amplitudes of harmonics were relatively smaller than the increase in the amplitude of FW, with a significant increase in the percentage that FW constitutes in the total amplitude of the pulse pressure waveform [13].

We suggest that HHC deserves further investigation because of its potential clinical utility and the paucity of investigations available. The HHC decrease with rising ICP can again be attributed to the decrease of the content of higher harmonics, which eventually causes the HHC to decrease.

Finally, the main source of error in the recording of mean ICP is zero drift of the micro-transducer. HHC is not affected by the zero drift therefore it may be useful in the detection of intracranial hypertension irrespective of the drift of the ICP micro-transducer.

### Cerebral autoregulation

Our data show that HHC is not correlated with the cerebral autoregulation, assessed with the PRx, in the entire monitoring period. The only statistically significant finding was a negative correlation between HHC and PRx during plateau waves, which probably indicates worsening of the autoregulation with decreasing compliance. We conclude that cerebral autoregulation and compliance are not directly linked and represent independent markers of cerebral function which can be variably impaired after brain injury.

### Limitations of the study

The study population consisted of patients with plateau waves and did not include all patients with ICP elevated above a certain threshold (e.g., 22 mm Hg). Although it can be seen as a limitation, intracranial hypertension is usually treated rapidly in clinical settings [9, 15, 17, 19, 26, 37], preventing the recording of lasting periods of eminent rise in ICP and studying its waveforms. Plateau waves, longer periods of elevated ICP in our material, provide an opportunity to study the features of intracranial hypertension. This is why they were chosen for this investigation. They are a peculiar phenomenon, mechanism of which has been explained by a model of vasodilatory cascade [32]. Other rises of ICP, e.g., due to brain oedema, contusion, acute hydrocephalus, and disturbance of venous outflow, do not share the same pathophysiological mechanism. Therefore, pooling them to assess the reaction of HHC seemed not to be justified and was not the aim of this study.

It is necessary to take into account that the selected group of patients under study may have tended towards those with prominent intracranial hypertension. However, in this work, we focused on the analysis of plateau waves as an interesting and well-described phenomenon triggered by active vasodilatation and increase in cerebral blood volume leading to elevation in ICP and decrease in CPP. These changes in cerebral blood volume, along with the reduced intracranial compliance, are known to be associated with changes in the shape of the ICP pulse waveform [8, 10], which, in turn, are reflected in changes in HHC. We also know from Berdyga et al. study [5] that HHC changes as a result of augmentation of intracranial hypertension during infusion test in patients with hydrocephalus. Our future studies should include all patients in whom ICP rises via other mechanisms and further investigate drifts in HHC in all physiological patterns of the intracranial hypertension.

It is also important to take into consideration the fact that an analysis of ICP elevated above a certain threshold could mask the observation of the presence of breaking point on the ICP-HHC characteristic in the current study. Further research on a larger number of patients will allow us to answer the question of what is the significance of the breaking point and whether it occurs for ICP increases above the assumed threshold (e.g., 22 mm Hg) or it is specific for ICP plateau waves only.

## Conclusions

This study supports the importance of the computerized analysis of the ICP pulse waveform. The HHC decreases at high ICP values. A decrease in the HHC may be a strong descriptor of intracranial hypertension. Detection of changes in the HHC may also help identify a significant zero drift of the ICP transducer. Further work should focus on relations between centroids’ shifts and clinical outcomes following TBI.
